# L2L: a simple tool for discovering the hidden significance in microarray expression data

**DOI:** 10.1186/gb-2005-6-9-r81

**Published:** 2005-08-31

**Authors:** John C Newman, Alan M Weiner

**Affiliations:** 1 Department of Biochemistry, University of Washington, Seattle, WA 98115, USA

## Abstract

L2L is a database consisting of lists of differentially expressed genes compiled from published mammalian microarray studies, along with an easy-to-use application for mining the database with the user's own microarray data. As illustrated by re-analysis of a recent study of diabetic nephropathy, L2L identifies novel biological patterns in microarray data, providing insights into the underlying nature of biological processes and disease. L2L is available online at the authors' website [].

## Abstract 2

A database with lists of differentially expressed genes from published microarray studies is presented together with an application for mining the database with the user’s own microarray data, allowing the identification of novel biological patterns in microarray data.

## Rationale

In only a few years since their development, high-throughput, whole-genome DNA microarrays have become an invaluable tool throughout biology. The appeal of microarrays seems most irresistible when the biological problem is most intractable; microarrays have become perhaps the most popular contemporary tool for hypothesis generation. Yet interpreting the mountain of data produced by a microarray experiment can be a frustrating chore. The most common outcome of such an experiment is a list of genes, or many such lists: genes that are induced or repressed under one condition or another, at one time point or another, in one cluster or another. The daunting task is to extract some meaning from these lists, either by identifying 'critical genes' which might single-handedly produce a biological effect, or by finding patterns in the list that point to an underlying biological process. The latter universally involves annotating each gene on the list and looking for groups of genes that share a particular characteristic. Until recently, this was done entirely by hand. Each gene was assigned, after a laborious literature search, to an arbitrary functional category like 'DNA repair' or 'metabolism'. A hypothesis might be based on which arbitrary categories appeared most often. Like any non-systematic approach, this one is vulnerable to our very human knack of seeing whatever pattern we wish in a noisy field. The Gene Ontology (GO) consortium [1] has brought systematic order to the field of gene annotation by pre-categorizing genes by biological process, molecular function, and cell component - thus eliminating the pattern-creating risk of *post hoc *annotation. A number of software tools now exist to automate the process of annotating a list of genes with GO categories. Several of these, including EASE [2], GOMiner [3], Onto-Express [4] and GO::TermFinder [5], also calculate the over-abundance of each category in the list, along with its statistical significance. However, even after functional annotation of the list of genes, uncertainty remains as to whether the results advance understanding of the biology at work in the system, and, if the system is a complex disease, whether the results help explain why the gene expression changes occurred. An alternative approach to interpreting gene expression data is to compare it with other related (or potentially related) gene expression data. The motivation is that microarray experiments exhibiting common changes in gene expression are likely to share one or more underlying molecular mechanisms. Furthermore, in some experiments, the underlying cause of the gene expression changes is well-defined: a specific gene deletion, for example, or treatment with a single receptor ligand. In such cases, the ability to connect the user's experiment with gene expression changes caused by a well-defined perturbation may lead immediately to a hypothesis regarding the underlying mechanism in the system under study.

**Figure 1:**
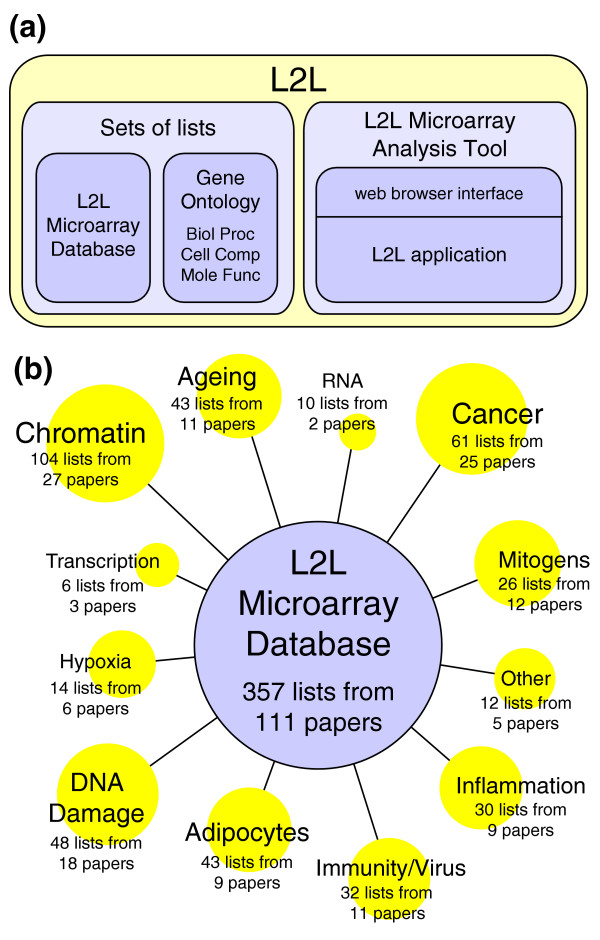
L2L and the L2L microarray database. **(a) **The centerpiece of L2L is the L2L Microarray Database, a collection of published microarray data in the form of lists of genes that are up- or downregulated in some condition. The L2L Microarray Analysis Tool (MAT) is a program that compares those lists with a user's microarray data, and reports statistically significant overlaps. The analysis tool includes a web browser interface, but the L2L application itself can be downloaded and run directly from the command line for batch or customized analyses. Three additional sets of lists, based on the three organizing principles of Gene Ontology, can also be used with the analysis tool. **(b) **The L2L Microarray Database contains over 350 lists compiled from over 100 selected microarray publications. A wide variety of topics are represented, from chromatin modifications and DNA damage to the immune response and adipocyte differentiation.

L2L is a database and associated software tool (Figure 1a) that systematically compares the user's own list of differentially expressed genes with a database of lists of differentially expressed genes that were derived from published microarray data, with the goal of finding common expression patterns that can help generate new hypotheses. The L2L Microarray Database was culled from 111 selected publications, and contains 357 lists of genes that were found to be either upregulated or downregulated under a particular experimental condition. The conditions represented in the database range from normal ageing to space flight, and from interferon treatment to histone deacetylase inhibition (Figure 1b). The L2L Microarray Analysis Tool compares each list in the database with a list of genes supplied by the user, and reports the statistical significance of any overlap between them. It also annotates each gene on the user's list with all the lists in the database on which it is found. The results are presented as a set of hyperlinked HTML documents, which can be conveniently explored by surfing from list to list and gene to gene. L2L is available as an easy-to-use online tool [6], and as a downloadable, command-line application released under the GNU General Public License.

## L2L Microarray Database

The need for a standardized format for presenting and storing microarray data from disparate platforms has been recognized for several years. A consortium of researchers [7] has detailed a standardized format for presenting microarray data (MAIME) [8] as well as a markup language in which to encode those now-standardized data (MAGE-ML) [9]. The data can be deposited in any of a number of large public repositories, including CIBEX, ArrayExpress, Oncomine and the NIH's Gene Expression Omnibus (GEO) [10-13]. All of these include web-accessible data-mining tools for browsing experiments and searching for the expression results associated with a particular gene. The sheer volume of deposited data is staggering, and represents a gold mine for bioinformaticians. Yet it all remains remarkably inaccessible to lay biologists. Although we can search GEO, for example, for microarray-identified genes one-by-one, there is no simple way to compare our data *en masse *with any other data in the repository, much less against all the data in the repository. Furthermore, repositories can make it difficult to extract the original results from the mass of deposited data; an interested user is often required to essentially re-analyze the data, with little knowledge of the original data analysis protocol or, in some cases, without access to all of the relevant data (for instance, GEO submissions do not usually include Affymetrix test-statistic data, a qualitative 'change call' which can be more accurate than the quantitative fold-change for detecting differential expression [14]).

The L2L Microarray Database collects an interesting subset of this public data in its most essential and accessible form - simple, well-annotated lists of genes, using a universal identifier, which were found to be either upregulated or downregulated under a particular condition. It is not intended to be an alternative to the public repositories, but an accessible and utilitarian supplement. The database can be easily applied to the global analysis of any gene expression experiment, producing insights that go well beyond gene-by-gene annotation. The development of L2L was inspired by our efforts to extract meaning from our own microarray analysis of the progeroid Cockayne syndrome (Newman JC, Bailey AD, Weiner AM, unpublished data), so the publications included in the database initially reflected topics thought to be related to this disease - ageing, cancer and DNA damage. Since then, the scope of the publications we included has expanded considerably to include chromatin structure, immune and inflammatory mediators, the hypoxic response, adipogenesis, growth factors, cell cycle regulators, and others. In spite of the parochial origins of the database, the wide range of topics now covered will make L2L of general interest to any investigator using microarrays to study human (and more generally, mammalian) biology. We demonstrate the breadth of L2L's utility below, by re-analyzing a published microarray dataset from a study of diabetic nephropathy - a subject completely unrelated to our original interests. Newman JC, Bailey AD, Weiner AM: manuscript in preparation.

## A good list is hard to find

We faced two major challenges in the creation of L2L, one philosophical and one practical. The philosophical problem, which has prevented any significant effort in this direction to date, is that no two microarray experiments are ever perfectly comparable. There is an almost infinite combinatorial complexity of organism, tissue type or cell line, RNA isolation technique, microarray platform, scanning instrument, experimental design, and data analysis technique - even if the question being asked is identical. To make a tool like L2L even possible, it is essential to exclude any incomparable information from each experiment, and convert the remainder to a common language that can be shared by all included experiments. We therefore removed all references to platform-specific probe identifiers, primarily because these would limit L2L to comparing experiments performed on identical platforms, but also because many manuscripts do not report probe IDs. Instead, we converted the probe IDs to the HUGO-approved symbols [15] of the genes they each represent, according the manufacturer's annotations, and ignored those that have no gene association because these cannot be reliably compared across platforms. We also excluded the reported magnitude of expression changes, because fold-changes are often not comparable across platforms [16]. Furthermore, fold-change can be a misleading indicator of the significance of expression changes, especially for platforms like Affymetrix GeneChips that use an independent, and more robust, change call calculation [14]. Finally, ignoring fold-changes vastly simplifies the computational task of comparing hundreds or thousands of lists.

The practical challenge was the extraction of published data and conversion to HUGO gene symbols. This was by far the most time-consuming of the tasks required to create L2L, despite the liberal use of automated tools. The first hurdle was the difficulty of extracting data from published papers in a usable form. Many tables of genes are published as graphical figures rather than textual tables. Supplemental data is often in the form of HTML tables, rather than text files. In both cases, the data are easy to view, but difficult to extract for other uses. More willful is the use of digital-rights management by certain journals to frustrate copying of any information from the electronic (PDF) version of the paper. In all of these situations, laborious manual transcription was required, instead of simple keystrokes to cut-and-paste the data. Repositories like GEO are only a partial solution to this presentation problem; the repositories contain all the raw data, but often lack information about the data analysis used to define a robust change, as well as the actual lists of robustly changed genes.

The second hurdle was actually identifying the genes on published lists. Many publications do not provide an unambiguous reference for each gene - only a common name and/or description. Those that do provide unambiguous references do so in a variety of forms - a HUGO name, LocusLink ID, GenBank accession, or (rarely) commercial probe ID. Online tools exist to interconvert many of these [17,18] and were used whenever possible to convert each list to HUGO names. Ambiguous references were hand-converted by finding the proper match in LocusLink or EntrezGene. Some lists in the L2L Microarray Database are derived from mouse experiments; these were first converted to standard mouse gene names, then mapped to the corresponding HUGO gene name using the HomoloGene database [19] with an *ad hoc *tool. Any genes without HomoloGene entries were matched by hand in EntrezGene to the proper human homolog. Any gene reference, mouse or human, which could not be unambiguously mapped to a HUGO name was ignored. Duplicates within a list were also ignored. The fraction of the original data that could eventually be mapped to a HUGO name varied with the quality of the gene reference, the proportion of expressed sequence tags (ESTs), and whether mouse-human conversion was required. Most datasets with unambiguous human references have greater than 90% of non-EST, non-duplicate gene references represented in the L2L list of HUGO names. Mouse-human conversion reduced this proportion somewhat (largely due to immunity-related genes), as did descriptive gene references (due to ambiguity). Each list in the database is annotated with a meaningful short name, a longer description, the platform used to generate the list (for example, Affymetrix U95Av2), one or more keywords, and the PubMed ID of the source publication.

## More than just microarray data

In addition to the L2L Microarray Database, L2L includes a set of lists for each of the three organizing principles of Gene Ontology - biological process, molecular function and cell component. These lists were compiled from the July 2004 GO association tables, which include associations between UNIPROT names and GO terms. UNIPROT's flat-files associate many human UNIPROT entries with a HUGO alias; an *ad hoc *tool was used to extract these relationships and convert the UNIPROT GO term assignments to unique HUGO GO term assignments. Another *ad hoc *tool then created a list for each GO term that contained every HUGO name associated with either that term or any of its descendants. Any lists with fewer than five genes were discarded because comparison to such a small list is unlikely to be informative. In all, there remained 2,169 GO-derived lists with a total of about 240,000 annotations, divided among the three organizing principles. A more detailed description of how the GO lists were compiled, along with downloadable versions of the *ad hoc *tools, is available on the L2L website [6].

Finally, L2L is not limited to using the four included sets of lists: L2L Microarray Database, GO: Biological Process, GO: Molecular Function, and GO: Cell Component. The modular nature of the tool means that new sets of lists can be created from any source of gene annotations. Some examples include protein-protein interaction databases like DIP, BRITE or BIND [20-22]; pathway annotations from KEGG, BioCarta or GenMAPP [23,24]; experimental gene expression modules [25]; or the associations of gene names with literature keywords that can be compiled using tools like PubGene and TXTGate [26,27]. Any source of gene annotation that can be represented as a set of lists, each specifying a group of genes that share some characteristic, can be easily used with L2L. We hope that the simple and open file formats will encourage others to contribute their own sets of lists to augment L2L or to create similar platform-independent resources.

Although we designed L2L for the lay biologist, we hope that the L2L Microarray Database will prove to be a valuable resource for the bioinformatician as well. For example, many investigators are interested in mapping networks of gene coexpression relationships with the goal of inferring previously unknown functional relationships, or even physical interactions, from shared expression profiles [28-30]. The L2L database is a significant source of primary data for such coexpression analyses. It currently contains 28,026 data points derived from microarray experiments, each of which represents a significant gene expression change. These data points encompass 10,151 gene names - a substantial fraction of the 33,000 HUGO names that had been assigned at the time of writing - and 6,009 of these genes occur at least twice in the database. Among these genes, there are 258,461 unique positive coexpression relationships (a pair of genes found together on different lists) that are found on at least two, and in some cases as many as 16, different lists. There are 20,338 negative coexpression relationships (pairs of genes that are inversely regulated, that is, one appearing on the 'up' and the other on the 'down' list for the same condition) that are found in at least two, and as many as ten, different conditions. We believe the L2L database's catalog of co-expression relationships is one of the largest yet available for human genes, and is based on more robust expression changes and a broader set of experimental conditions than other, albeit more sophisticated, efforts [31].

## L2L microarray analysis tool

**Figure 2:**
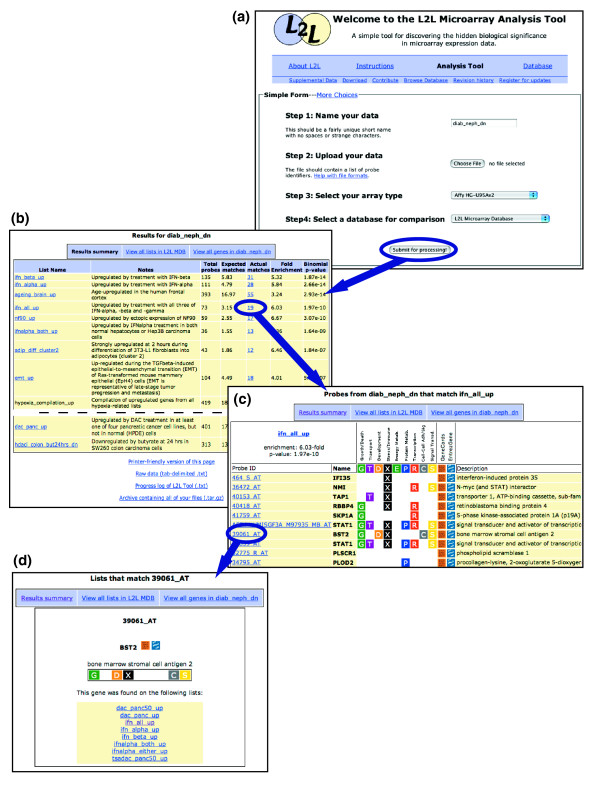
L2L uses a simple web-based interface, and generates easy-to-navigate, annotated HTML pages as output. **(a) **The L2L web interface. **(b) **The Results summary page displays each list from the database that significantly matched the data, along with links to list annotations and Listmatch pages. **(c) **An example Listmatch page, which displays all of the probes on a list that match the data, with a variety of annotations and links to Probematch pages. **(d) **Probematch pages show all of the lists on which a probe is found, with links back to their Listmatch pages. Arrows indicate sample navigation paths between the output pages.

Compiling the L2L Microarray Database took a large investment of effort that we are eager to share with the community. The open file format of the L2L lists can be easily adapted for use in existing list-comparison tools, like EASE [2] and VennMapper [32]. We saw a need, however, for a similar general-purpose tool that was as straight-forward to use as, for example, PubMed Entrez, and which could be optimized for presenting the unique sort of relationship data contained in the database. Therefore, we created the L2L Microarray Analysis Tool - simple to use for the lay biologist, while powerful and customizable for the technically inclined. Upon entering the L2L website [6], the user follows four steps - step 1: enters a name for the analysis, step 2: uploads a data file, step 3: selects the microarray platform from a menu, and step 4: chooses which set of lists will be used to analyze the data (the database or one of the GO sets) (Figure 2a). After L2L has finished comparing the user's data with all the selected lists, it creates a set of easy-to-navigate HTML pages to visualize the results. These are of three types: the Results Summary page, Listmatch pages and Probematch pages. The Results Summary (Figure 2b) displays all of the lists that have a statistically significant overlap with the user's data, along with all relevant statistics. Each list has a unique Listmatch page (Figure 2c), which displays all the probes in the data that matched that list, along with a variety of annotations for each probe. Similarly, each probe in the data has a Probematch page (Figure 2d), which displays all the lists on which that probe was found. The pages are interconnected by hyperlinks, making it easy to surf, for example, from the Results Summary to a list, to a gene found on that list, to a different list on which that gene is found. Lists and genes are described briefly on each page, but are also hyperlinked to external annotations: for the database lists, this is usually the PubMed abstract of the source publication; for GO categories it is the AmiGO browser page [33] for that category; for genes it is the GeneCards [34] and EntrezGene [35] entries. From the Results Summary page, all of the output files can be downloaded by the user, and viewed later with any web browser.

**Figure 3:**
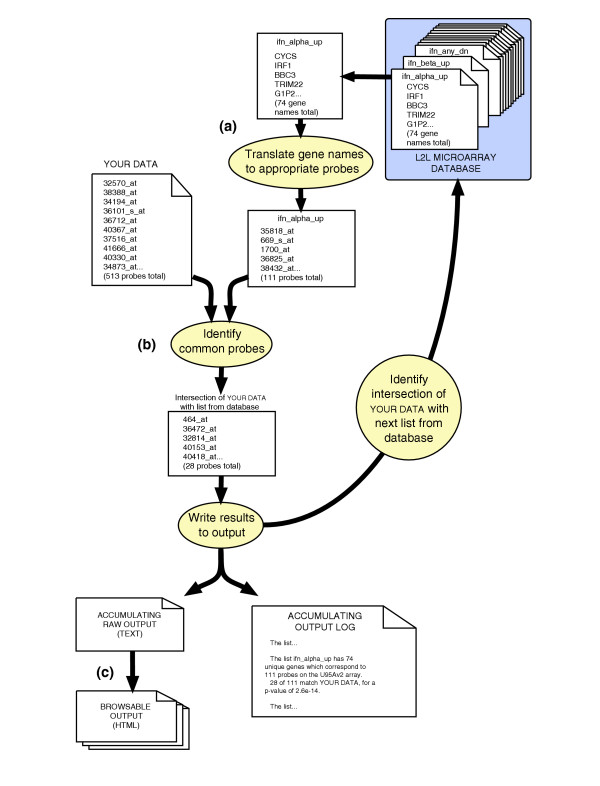
The L2L application sequentially compares each list in the database with the input data, and records the overlap between the two lists of genes. **(a) **Each list in the database is a list of HUGO symbols. These are first translated to the corresponding microarray probes that represent those genes. Depending on the microarray, some genes on a list are represented by multiple probes and some by none at all. **(b) **The program finds the intersection between the translated list of probes from the database and the user's list of probes. The results are logged and written to a raw output file. The program then proceeds to the next list in the database. **(c) **Once all lists in the database have been compared with the user's data, the program creates a set of HTML pages to browse the output.

The analytic engine of L2L is the L2L application, written in Perl (Figure 3). This program receives user input from the web interface and performs the actual data processing tasks, along with the creation of the output HTML pages. The program requires three inputs: the data to be analyzed, in the form of a list of microarray probe identifiers; a translator library that pairs each probe on the microarray with its corresponding HUGO gene name; and a folder of lists with which the data will be compared. As described above, these lists are in the form of HUGO gene names. The program works sequentially through all the lists, first using the translator to map each gene name in the list to all the probes on the microarray that represent that gene (Figure 3a). Each of these translated probe IDs is then queried against the data. Thus, a given gene on a list may be represented by several microarray probes, or none at all. This name-to-probe translation - the reverse of the process by which the database lists were originally generated - allows L2L to retain the greatest possible amount of the user's data, by performing comparisons based on the probe IDs of the user's microarray, rather than the gene names those probes represent. The loss of this probe ID information from the database lists was an unfortunate necessity, since relatively few studies from which the database was compiled even reported probe IDs. The retention of probe IDs from the user's data allows some expression of the subtleties that multiple probes per gene can afford. If only one splice form of a gene is upregulated in the user's data, only that one probe will be scored as a match to a database list the gene is on; all other probes for that gene will be queried and counted as non-matches. The program records the number of probes derived from the list that match the data, the total number of probes on the microarray that represent the gene names on the list, and the fraction of probes on the microarray that are found in the data (Figure 3b). From these three numbers, the program first calculates the number of expected matches for that list, then the relative enrichment of actual matches, and finally a *p *value for the significance of the overlap. The *p *value represents the cumulative probability of finding at least as many matches between the data and the list, given the fraction of all microarray probes that are found in the data, as calculated with a cumulative binomial distribution (see below for a more detailed discussion of the statistics of L2L). The results are logged and written to a raw output file. In addition, for each list, the program records the IDs of all the probes from the data that matched that list. Similarly, for each probe in the data, the program records the names of all the lists on which it was found. All of this information is then used to create the output HTML pages (Figure 3c).

The modular design of L2L means that there are a variety of ways to interact with the L2L application, depending on the user's needs. The simplest is through the web interface. In addition to the four-step form described above, there is a 'More Options' page that allows the user to upload a custom translator library for microarray platforms that are not on the menu. Thus, while L2L is intended primarily for use with whole-genome expression microarrays, it can be used with data from any genomic or proteomic analysis. Alternatively, the L2L application itself can be downloaded and run from the command line on any computer with Perl and a UNIX-like command shell. This is ideal for users who want to use a custom set of lists or who need to rapidly process many different data files in a batch mode. L2L includes a basic textual interface that prompts the user for the location of the three necessary inputs: data file, translator library and set of lists. A batch mode bypasses the interface and allows the processing of any number of data files, each from a different microarray platform, against any or all sets of lists with a single command. Users are also free to download the entire L2L website and run it on their own web server.

L2L is remarkably fast because all of the potentially billions of search-for-match operations are implemented as hash-table lookups in Perl. Since relatively few data are stored in memory at any one time, performance is processor-bound on modern machines, and scales linearly only with the combined size of the lists - not with the size of the data file. A comparison of virtually any size data file to all 357 lists in the database, along with the creation of all output files, takes only about 15 seconds on a 1.4 GHz PowerPC. All files associated with L2L, including data, translator library and list, are in a simple tab-delimited, flat-file format. A detailed description of each file type is available on the L2L website [6]; users can create their own files from any text editor.

## L2L in the real world: diabetic nephropathy

The ultimate test of a utility like L2L is whether it can produce novel biological insights from real-world microarray data. With this objective in mind, we downloaded several publicly available datasets and analyzed their lists of gene expression changes with L2L (the sample datasets and all results are available at the L2L website [6]). Diabetic nephropathy (DN) is one of the most common, and most devastating, complications of type 2 diabetes mellitus (T2DM) but its molecular etiology remains poorly understood. To generate new hypotheses, Baelde and colleagues examined gene expression patterns in human kidney glomeruli isolated either from normal kidneys or from kidneys afflicted with DN [36]. Several hundred genes were found to be significantly changed in DN, and these were then classified according to GO category using MAPPFinder [37]. The primary hypothesis that ultimately emerged from the experiment, however, relied entirely on an analysis of 'critical genes' - a handful of genes with biological functions that seemed likely to be relevant. Specifically, dysregulation of several tissue repair genes and repression of the growth factor VEGF led the authors to suggest diminished repair capacity in capillary endothelium as a possible etiology for DN. They also suggested, based on MAPPfinder's list of overabundant GO categories, that DN kidneys suffer from reduced nucleotide metabolism and disturbed cytoskeleton formation.

**Figure 4:**
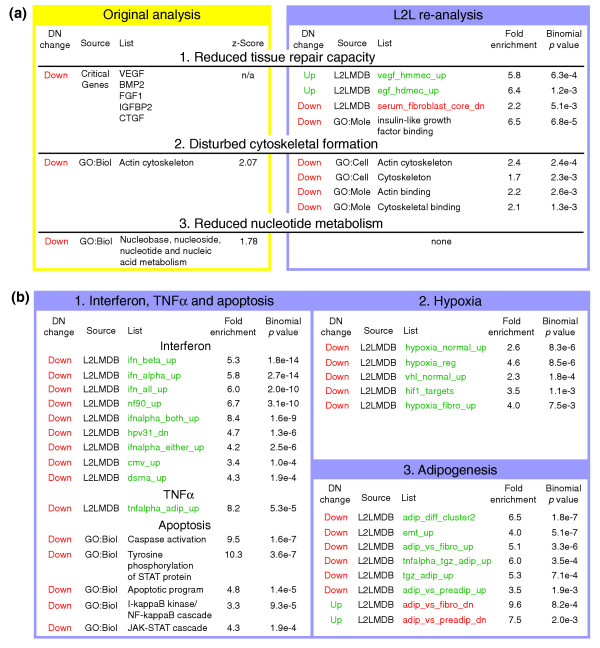
L2L analysis of gene expression changes in diabetic nephropathy (DN). **(a) **Three major conclusions of Baelde *et al. *[36] revisited. L2L finds support for cytoskeletal dysfunction, but no evidence of reduced nucleotide metabolism. Evidence for the central thesis, reduced tissue repair capacity, is mixed. L2L found reduced expression of IGF-binding proteins, suggesting a defect in response to these growth factors. However, L2L also found a correlation between genes repressed by the serum-response and genes downregulated in DN, as well as a correlation between genes upregulated in DN and genes induced by EGF and VEGF - despite reduced expression of VEGF itself in DN kidneys. **(b) **Three new biological themes in DN found by L2L. 1. Interferon, TNFα, and their associated apoptotic pathways are all downregulated in DN. 2. The hypoxia response is impaired in DN. 3. Pathways associated with adipogenesis and adipocyte function are downregulated in DN. Complete results, along with descriptions and annotations for all lists, can be found on the L2L website [6]. Red or green denote reduced or increased expression, respectively, in DN or in the condition represented by a list.

Analysis of the same data with L2L not only quickly confirmed some of the authors' conclusions (Figure 4a), but also detected the fingerprints of the underlying disease process (Figure 4b). Using L2L with Gene Ontology lists, we confirmed the finding of disturbed cytoskeletal formation within moments. We also found that genes repressed in DN are enriched for genes that function in apoptotic pathways involving JAK-STAT, IκK-NFκB and caspases, as well as IGF-binding proteins. Although the latter evidence for a reduced insulin-like growth factor response appears to support the authors' central hypothesis, comparison of the DN data with the L2L Microarray Database produced contrary evidence. We found a correlation between genes upregulated in DN and the response to serum, EGF and VEGF. The observation that glomerular cells express higher levels of growth factor target genes in DN than in normal kidneys suggests that DN kidneys may be coping adequately with lower VEGF expression. The molecular etiology of DN may, therefore, lie elsewhere.

Three novel themes emerged from the comparison with the L2L Microarray Database of genes downregulated in DN. Firstly, many of these genes are induced by interferon - nine lists related to interferon and the viral response overlap very significantly with the list of genes repressed by DN (*p *values from 2e-4 to 2e-14). Perhaps related to this, genes downregulated in DN also significantly overlap with genes induced by tumor necrosis factor (TNF)α (*p *= 5e-5). Secondly, hypoxia-induced genes are repressed in DN - five lists have *p *values from 8e-3 to 8e-6. Thirdly, and most surprisingly, five lists of genes upregulated in adipocyte differentiation and function overlap with genes repressed by DN (*p *values from 2e-3 to 2e-7), whereas two lists of genes downregulated during adipocyte differentiation correlate with genes upregulated in DN (*p *= 0.002 and 0.0008).

The relationship between genes repressed in DN and genes induced by interferon (IFN) illustrates an important caveat regarding tissue-based microarray experiments: the complexity of the tissue itself makes it difficult to determine whether the results reflect changes in expression within glomerular cells, a different degree of leukocyte contamination, or even changing gene expression within those leukocytes. The latter two scenarios are consistent with previous findings of dysfunctional cell-mediated immunity in diabetes [38-41]. The association of genes repressed by DN with those induced by TNFα may be interpreted in this context as well, because at least one study suggested poor response to TNFα as one reason for the immune deficiency in T2DM [39]. Since no cytokines appear on the list of differentially expressed genes, these data suggest - supposing the gene expression changes reflect contaminating leukocytes - that a poor transcriptional response of leukocytes to cytokines may cause the immune deficiency in T2DM.

The most widely accepted theory of pancreatic β-islet cell dysfunction in T2DM is that a variety of inflammatory signals from diet, adipocytes and the immune system combine to trigger apoptosis in those cells [42,43]. Two of the most important signals are thought to be TNFα from adipocytes and IFNγ from leukocytes. It is intriguing, therefore, that while the L2L analysis found downregulation of IFNγ- and TNFα-induced genes in DN, the GO:Biological Process analysis specifically identified the downstream apoptotic effectors of these two cytokines (JAK/STAT for IFNγ, IκK/NFκB for TNFα) as also downregulated in DN. So rather than being an artifact of leukocyte contamination, these results could reflect reduced sensitivity to the blood-borne inflammatory signals that, in sensitive pancreatic islets, trigger β-islet cell apoptosis - the hallmark of the underlying disease.

The second theme - a poor hypoxic response - suggests a transcriptional defect more specific to glomerular cells. At first glance, the direction of this correlation is surprising: DN kidneys should already be under hypoxic stress if poor angiogenesis and endothelial dysfunction are partially responsible for DN. However, this effect is apparently swamped by the ischemia experienced by all kidneys following extraction, before RNA is harvested. Although all kidneys were handled identically, hypoxia-response genes were more strongly induced in the normal controls. This could suggest that DN glomeruli are already stressed, and unable to respond fully to further stress. The result could be a downward spiral of increasing damage and reduced function.

Adipogenesis, the third theme, also seems puzzling at first. Why would adipocyte differentiation genes be differentially regulated in kidney glomeruli? Another hallmark of diabetes is deranged adipocyte function - adipocytes are insulin-resistant, have diminished capacity to store fat, and secrete excessive amounts of inflammatory cytokines and free fatty acids [44]. Such dysfunctional adipocytes may be primarily responsible for creating the chronic inflammatory state that brings about overt disease [45]. Adipocytes are also one of the primary targets of the most widely used class of antidiabetic drugs. Thiazolidinediones (TZDs) are agonists of PPARγ, a transcription factor required for early adipocyte differentiation. TZDs can help restore normal adipocyte function in diabetics [46]. The dysregulation of adipocyte differentiation genes, therefore, may be another fingerprint of the underlying disease, indicating either the dysfunction of contaminating adipocytes in the glomeruli preparations, or a surprising sensitivity of glomerular cells to the same dyslipidemic signals that perturb adipocyte function in diabetics. Interestingly, a microarray analysis of a mouse model of DN, contemporary with this human study, found deregulation of a number of lipid homeostasis genes [47].

Taken together, the L2L results demonstrate the importance of considering T2DM and its complications as part of a single, integrated disease process. The fingerprints of the underlying disease - inflammatory factors and adipocyte dysfunction - are readily detectable in kidney glomeruli, and suggest that the same factors that cause β-islet cell and adipocyte dysfunction are responsible for glomerular dysfunction as well. In fact, PPARγ is expressed in rodent glomeruli [48,49] and treatment with a TZD enhances renal function in both rats and humans [50-52]. It would be interesting to determine which dyslipidemic signals affect DN glomeruli; how those signals are transduced in glomerular cells; and whether the result is abnormal intracellular lipid accumulation [47], or direct inhibition of glomerular function by activation of specific intracellular signaling pathways [50] - either of which might prevent glomerular cells from responding to normal growth and stress signals.

## L2L and the genomics of ageing

Deregulation of gene expression is now thought to underlie many of the effects of ageing in a variety of organisms, including humans. There is a well-defined link between human ageing and disruption of normal DNA methylation patterns [53-55]. A 'unified theory of ageing' has even been proposed, which asserts that 'the progressive and patterned alteration of chromosome structure is the primary cause of ageing' [56]. Other investigators have suggested that such transcriptional deregulation is a programmed response to stresses that increase with age [57], the stochastic result of failed genome maintenance [58], or the specific result of the disruption of some critical (but unknown) cellular function [59,60].

We analyzed two recent gene expression studies of the ageing human brain, to see if there were common patterns in the transcriptional deregulation. Lu and colleagues [61] found significant gene expression changes in the frontal cortex of individuals from 26 to 106 years of age. Genes involved in synaptic plasticity, vesicular transport and mitochondrial function were downregulated, while stress-response, antioxidant and DNA repair genes were upregulated. They found increased DNA damage at the promoters of downregulated genes, leading them to suggest that 'DNA damage may reduce the expression of selectively vulnerable genes involved in learning, memory and neuronal survival, initiating a programme of brain ageing that starts early in adult life'. Blalock and colleagues [62] correlated hippocampal gene expression with histological and clinical markers of Alzheimer's disease (AD). They found a large number of genes whose expression changes correlate with either or both incipient and overt disease, and suggest that the pathogenesis of AD is 'genomically orchestrated'. EASE analysis [2] showed that growth, differentiation and tumor suppressor pathways are upregulated early in the disease process, while protein-processing pathways are downregulated.

Using Gene Ontology lists, L2L quickly replicated the EASE results of Blalock *et al. *(the complete analysis is available on the L2L website [6]). Using the L2L Microarray Database, L2L also revealed a novel link between AD and the hypoxia response. Genes upregulated with overt AD overlapped significantly with two lists of genes upregulated in myocardium during heart failure (*p *values 2e-5 and 8e-10) and three lists of genes specifically induced by hypoxic stress (*p *values 0.002 to 0.005). Moreover, genes downregulated with overt AD overlapped with two lists of genes downregulated in heart failure (*p *values 0.004 and 5e-5).

**Figure 5:**
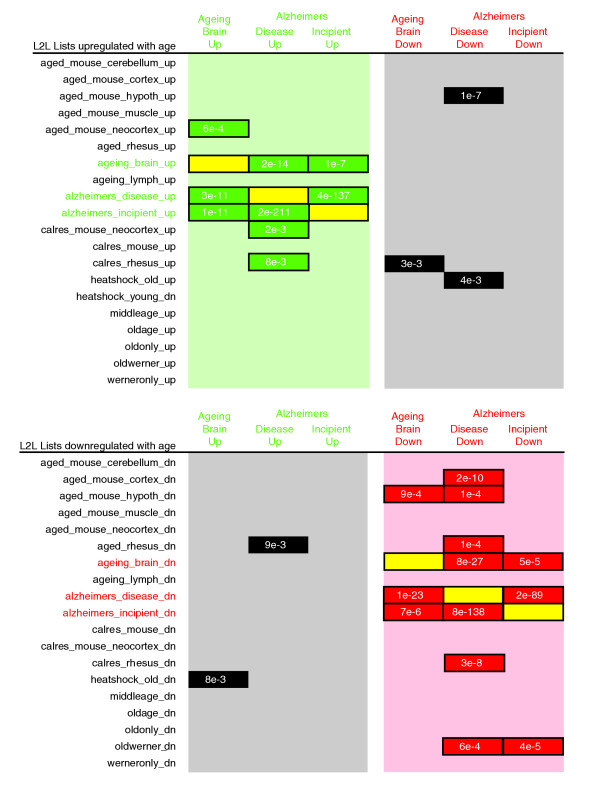
L2L analysis of gene expression changes in two studies of the ageing human brain. Lists of differentially expressed genes from Lu *et al. *(ageing_brain) [61] and Blalock *et al. *(alzheimers_disease and alzheimers_incipient) [62] were compared with all ageing-related lists in the L2L Microarray Database, including each other (all data are available on the L2L website [6]). Numbers represent binomial *p *values for significance of overlap. Green denotes overlap between lists of genes upregulated with ageing; red denotes overlap between lists of genes downregulated with ageing; black denotes overlap between lists of contrary directions; yellow denotes self-self comparisons.

Most intriguing, though, was that analysis of these two datasets with the L2L Microarray Database showed a surprisingly consistent overlap in gene expression with each other and with a variety of other ageing-related studies, suggesting that models of mammalian ageing exhibit a common transcriptional pattern (Figure 5). The database currently contains a total of 39 ageing-related lists, including the six lists derived from these two studies. Querying those six lists produced 29 instances of significant overlap (*p *< 0.01) with other ageing-related lists (encompassing 17 of the 39). Furthermore, 24 of the 29 overlaps were in the expected direction (up-up or down-down). In particular, the degree of overlap between these two datasets was dramatic. When the dataset of Lu *et al. *was compared with the database, genes upregulated in the ageing human brain overlapped very significantly with genes upregulated in incipient (*p *= 1e-11) and overt (*p *= 3e-11) AD. Conversely, genes downregulated in the ageing human brain overlapped genes downregulated in incipient (*p *= 7e-6) and overt (*p *= 1e-23) AD. Querying the database with the data of Blalock *et al. *produced similar results (*p *values ranging from 5e-5 to 8e-27), as well as demonstrating the enormous overlap between the incipient AD and overt AD datasets (*p *values from 2e-89 to 2e-211). Other significant overlaps were found with the progeroid Werner syndrome [60], caloric restriction in mice [63] and rhesus monkeys [64], ageing monkey muscle [64] and ageing mouse brain [65,66].

Although patterns of related gene expression changes were easily found in a variety of ageing models, we could not clearly define a set of age-regulated genes. A small group of genes was commonly regulated in the two human studies we examined, but none was also consistently regulated in studies of mouse or monkey models, or even in human studies of other tissue types. Indeed, when only those genes that are commonly regulated in human brain were queried against the L2L Microarray Database, no significant overlaps were found except with the studies from which they were derived. Taken together, these data suggest that while transcriptional deregulation is a fundamental feature of cellular ageing phenotypes, the detailed transcriptional profiles are tissue-specific and perhaps, to some degree, stochastic. Thus, ageing-related gene expression changes in different tissues and models are sufficiently similar to suggest a common underlying mechanism, perhaps DNA damage to sensitive promoters [61] or failure to maintain chromatin structure [67]; however, differences between the profiles suggest that the specific genes deregulated in each situation must be drawn from a larger pool of genes exhibiting varying degrees of vulnerability to deregulation. This illustrates both the danger of relying too heavily on a 'critical genes' approach to explain ageing phenotypes, as well as the hope that there may well be a common underlying mechanism of transcriptional dysregulation waiting to be elucidated.

## Reliability of L2L results

The question remains as to whether the results of an L2L analysis can be trusted. These concerns fall into two major categories, which might be described as qualitative and quantitative. The qualitative concern is whether the lists of differentially expressed genes in the database are trustworthy, and if comparison to a user's data can be meaningful. The quantitative concern is whether the statistics we use to judge the significance of the overlaps between a user's data and lists from the database provide a useful metric of biological meaning.

Could a small amount of poorly analyzed or biased data in the L2L database poison the well for all who drink? Much like the scientific process as a whole, L2L takes a distributed-competence approach, augmented by independent replication and careful statistical analysis, to mitigate this concern. Our working assumption is that investigators themselves are best qualified to judge the quality of their own data, and that published lists usually include only those genes for which a change call can be assigned with a reasonable probability. We augment this assumption by including in the database, whenever possible, microarray datasets generated by independent groups that have addressed the same or a closely related question. Given the noise inherent in any microarray experiment, a user can feel much more secure interpreting results which reflect overlap with several related database lists from different sources, rather than idiosyncratic overlap with just one list. Finally, L2L calculates a *p *value for each comparison that provides a quantitative assessment of the significance of an overlap. If an experiment is contaminated with random data due to experimental error or systematic bias, the likelihood of the L2L list derived from that experiment overlapping significantly with any other experimental data would be purely stochastic - unless both experiments suffer from a common systematic bias. For example, we performed a 10,891-trial simulation with randomized data to help validate our sample analysis of diabetic nephropathy. The odds of achieving a *p *value below 0.05 with random data was no greater than 0.05 for any list in the database, and as low as 0.001 (see supplemental data on the L2L website [6]). In the absence of common systematic bias, therefore, random data are very unlikely to produce spuriously significant L2L results.

There are two major potential sources of systematic bias: genes that are considered *a priori *to be 'interesting' or 'critical' based on previous data or theory, and platform-specific bias. Certain often-studied, well-understood genes - the very kind that lend themselves to 'critical gene' hypotheses - are represented on virtually all microarray platforms, and thus could be more likely to be found in random data acquired with any platform. Certain genes may also be more likely to be flagged as differentially expressed on a particular type of chip, perhaps because the chip is more sensitive to small variations at particular expression levels or because of probe-specific effects. If any systematic bias exists, it could only represent a higher likelihood of a random change in signal for that gene or probe - the chip does not know whether the control or experimental RNA is washed onto it, or with which dye color. So proper experimental design and data analysis should eliminate these false-positives before a user turns to L2L. The same applies to the published data from which the L2L lists are derived. If any false-positive genes do persist on database lists, the fact that L2L separately analyzes 'up' and 'down' lists mitigates their impact, because they will be randomly distributed between the two lists. These separate lists also provide great potential assurance for the user, if the 'up' and 'down' lists in the user's data both correlate significantly and respectively with the 'up' and 'down' lists (or vice versa) for a particular condition in the database (see Figure 4b, diabetic nephropathy and adipogenesis). The inclusion of data from independent groups can provide further assurance, because the same set of randomly changing genes is unlikely to be found in independent datasets from different platforms. Still, both sources of systematic bias can be directly addressed in a future release of L2L by more sophisticated statistical analysis algorithms. Each list in the database is annotated with the platform that produced it, so the frequency of occurrence of genes among lists from a given platform (platform-specific bias) as well as the overall occurrence of genes in the database (bias toward 'interesting genes') could be used to weight the contribution of each gene match to the overall significance of the overlap between two lists.

## Statistical considerations

If we accept in principle that measuring the overlap between a user's list and the various lists in the L2L database can produce biological insights, we still must resolve how to quantify that overlap with a meaningful statistic that provides at least a relative gauge of which overlaps deserve the most attention. Three major considerations are the choice of statistic, the multiple-hypothesis problem, and the issue of *p *value inflation. We performed a variety of analyses on our sample dataset of genes downregulated in diabetic nephropathy in order to determine how well the relatively simple binomial distribution calculation performs under real-world circumstances. The results for a selection of 22 lists, those upon which we based our conclusions about diabetic nephropathy, are presented in Table 1. Complete results for all lists, and more detailed information about how these analyses were performed, are available on the L2L website [6].

The essential task for a statistical test in over-abundance analysis is to quantify how surprised we should be to see a particular degree of overlap or, conversely, how likely it is that the overlap occurred by chance. If the likelihood of success in a trial is *p *, and we perform *n *trials, what are the odds that we will see *m *or more successes? In the case of L2L, *n *is the number of probes that map to a list in the database, and *p *is the likelihood that any one of them will be found in the data by chance - the proportion of probes in the user's data out of all the probes on the microarray. A 'trial' tests whether one of the *n *probes derived from a database list is found in the user's data; success is a match. The binomial distribution permits the exact calculation of the odds of achieving a particular number of matches out of *n *trials. The cumulative probability of achieving *m *or more matches is found as follows:



L2L uses the Double Precision Cumulative Distribution Function Library (DCDFLIB) [68], implemented in the Math::CDF Perl module [69], to compute binomial probabilities. The binomial distribution performs trials with replacement - the odds of scoring a success remain constant for all trials. In reality, a probe can only be selected once, so the hypergeometric distribution, which calculates probabilities without replacement, is more accurate. However, it is more difficult to calculate than the binomial distribution, and in any event approaches the binomial distribution at large values of *n *and *m*, where replacement has little impact on the odds of the next trial. Alternatively, the Poisson distribution is easier to calculate than the binomial distribution, and approaches it where values of *n *are large and *p *small (as in most L2L analyses) [70]. In our sample dataset of genes upregulated in diabetic nephropathy, the *p *values calculated from the hypergeometric distribution or Poisson distribution closely followed those calculated from the binomial distribution (Table 1; compare columns 5, 6 and 7). We therefore chose to use the binomial distribution as a reasonable compromise between accuracy and computational requirements.

The multiple-hypothesis problem is that when testing a large number of hypotheses simultaneously - here, that each of the hundreds of lists in the L2L database might overlap significantly with the user's data - the odds of producing a low *p *value by chance become substantial [71]. For example, with 357 lists in the L2L database, we might expect purely random data to produce about 18 'significant' overlaps with *p *values <0.05 (357 * 0.05). There are two common approaches that either reduce the odds of seeing any such false-positive *p *values, or mitigate their effect. The former approach is to control the family-wise error rate, usually by applying some adjustment to the calculated *p *values. This adjustment can be the same for all *p *values (termed 'single-step') or can vary as we evaluate each *p *value in order ('step-down' or 'step-up'). The single-step Bonferroni is the most common adjustment, and is simply the multiplication of the *p *value by the number of hypotheses (p * *n*, *n *being 357 in this case). We found the Bonferroni adjustment to be excessively conservative, based on the simulation-adjusted *p *values and false discovery rate (see below, and Table 1). The single-step Sidák, which uses the adjustment (1 - (1 - *p *)^*n *), produced near-identical results to the Bonferroni for low *p *values. Since *n *has a large initial value, step-down procedures for these two adjustments - where *n *is decremented by 1 as we adjust each *p *value in ascending order - did not produce substantially different adjusted *p *values.

An attractive alternative to simple adjustments based on the number of hypotheses is to perform simulations with random data, and adjust *p *values based on their frequency of occurrence among the random results. We therefore undertook a 10,891-trial simulation using datasets of the same size as our diabetic nephropathy sample (513 probes), drawn randomly from all the probes on the U95Av2 microarray (10,877 probes). We used true random numbers from Random.org [72] for all simulations. As expected, the median binomial *p *value calculated from these random data was not significant for any list (Table 1, column 9). We compared each *p *value from the actual sample data to the simulation-generated *p *values for that specific list, and for all lists together. In both cases, the frequency of occurrence of a *p *value equal to or less than the actual *p *value (that is, the simulation-adjusted *p *value) was generally lower than the actual *p *value (Table 1). This shows that, at least for the diabetic nephropathy dataset on the U95Av2 platform, a simple calculation of *p *values based on the binomial distribution gives a good approximation of the actual likelihood of seeing an overlap by chance. The capability to perform a simulation analysis will be included in a future release of the downloadable L2L application. However, the utility of a simulation analysis is proportional to the number of trials run, because an adjusted *p *value cannot be lower than (1/number of trials). Each 'trial' is a full-fledged L2L analysis, so a 10,000-trial simulation takes four orders of magnitude longer to run than a single analysis, not considering the time required to create random datasets. The computational requirements are therefore daunting, and preclude it from being practical in a web-based tool.

All such *p *value adjustments, however they are made, aim to reduce the chances of seeing any false positives. They can therefore be too conservative if, as in most biological questions, permitting a few false-positives is a reasonable trade-off for seeing more true data. The false-discovery rate (FDR) is an increasingly popular approach to the multiple-hypothesis problem that mitigates the effect of false-positives by estimating how many there are at a given level of significance, rather than trying to eradicate them [73]. It can therefore be substantially more powerful than controlling the family-wise error rate. We used our random-data simulation to calculate the FDR at all levels of significance by dividing the average number of random occurrences of a *p *value less than or equal to a given number by the number of occurrences in the actual data of a *p *value less than or equal to that number. Column 12 of Table 1 shows that if we use the least significant binomial *p *value of our 22 sample lists (0.0075) as a cutoff, only 2% of the lists with equal or lower *p *values are expected to be false positives. Overall, a binomial *p *value of 0.05 corresponded to an FDR of about 10%, and 0.01 to 2.5%. The capability to calculate FDR from simulation data will be included in a future version of the downloadable L2L application, but these sample data suggest that the simple and economical binomial calculation of L2L, with a rough *p *value threshold of 0.05-0.01, strikes a reasonable balance between stringency and power.

Finally, we must address the issue of *p *value inflation: the generation of *p *values that, while genuinely statistically significant, are devoid of biological meaning. One way this can occur is through the statistics of small numbers - the anthropic principle of over-representation analysis. When only very few genes in the universe being tested possess a given characteristic, even one occurring in the data may be calculated as highly significant. Unlike a Fisher's exact test, the binomial distribution makes no explicit accommodation for small numbers. However, in creating L2L we assumed that comparisons with very short database lists would not be meaningful, and excluded lists (including those generated from GO annotations) with fewer than five genes. For a moderately sized dataset like our sample (513 genes), two out of five probes must match the data for a significant *p *value (0.01) to be generated. For much smaller datasets, only a single matching probe could produce a significant *p *value (for 50 genes, 0.02). However, the goal of L2L is to tease out complex patterns of gene expression that might be produced by a kaleidoscope of pathways. There is simply too small a signal among a few dozen genes to identify meaningful patterns, unless the investigator is certain that only a single pathway is at work - in which case L2L is unlikely to be helpful anyway. We therefore intend L2L to be used with relatively large database lists and relatively larger datasets, and in such circumstances the dangers of small numbers should be minor. We quantitatively tested the robustness of L2L's results by performing a 10,891-trial permutation simulation. In each trial, 52 probes from the sample data (10%) were thrown out and replaced with 52 different probes drawn randomly from the universe of the U95Av2 microarray. We found that the median *p *values generated by the permutations were only slightly reduced from the actual values. In no case was the actual *p *value a significant outlier among the permuted data: all had list-specific *p *values of >0.05, most with FDRs of 70-80% (Table 2).

The second potential source of *p *value inflation arises from the universal nature of the database. The common language, HUGO symbols, must be translated to platform-specific probe identifiers for the user's microarray. If only a handful of genes in a database list are represented on the microarray, and one of those genes happens to be represented by several probes, all of which are differentially expressed in the user's experiment, the list will generate a highly significant *p *value on the questionably narrow basis of that single gene. A user can see on a Listmatch page exactly which genes or probes created a small but significant overlap, and judge if it appears to be an artifact of translation. Users should be particularly wary of genes used as hybridization controls. We re-analyzed our diabetic nephropathy sample data without probe translation, using only gene symbols (Table 2). Several of the 22 sample lists dropped out of statistical significance; most of these were due to STAT1, an Affymetrix hybridization control, being represented by six probes in the data. Users may wish to remove control probes from their data before analyzing it with L2L. A future release of L2L will incorporate a directed-permutation algorithm into the statistical analysis to ensure that a reported *p *value is not overly reliant on a single gene.

## L2L is a unique microarray analysis tool

The idea of finding the overlap between two lists of differentially expressed genes, like the idea of a central repository of microarray data, dates to the earliest microarray experiments. One of its earliest expressions was through Venn diagrams that compare differentially expressed genes within a single series of experiments. Global clustering of microarrays is a more sophisticated, and more popular, example of this sort of comparative analysis [74], and has proven its worth for class discovery - for example, defining new, and potentially biologically relevant, subspecies of tumors [75,76]; and for class prediction - for example, predicting the behavior and susceptibility to therapy of a tumor by comparison to tumors with known outcomes [77,78]. However, the simpler pair-wise approach of L2L has the advantages of extending well across different platforms and not requiring access to raw data - only to lists of differentially expressed genes. It is well suited, therefore, to its task of finding common patterns between diverse gene expression studies, and enabling biological inferences to be drawn from the commonalities it finds.

VennMapper, created by Smid *et al. *, is one recent attempt in this direction [32]. It is a software tool that identifies overlaps in lists of differentially expressed genes (defined by an arbitrary fold-change cutoff) from user-supplied heterologous datasets, and calculates the statistical significance of the results using a z-value derived from a normal binomial distribution. The statistical approach is similar to that used by a variety of data mining tools that examine a list of genes for over-representation of GO categories, like GOMiner, EASE, Onto-Express and GO::TermFinder [2-5]. VennMapper and EASE, like the L2L Microarray Analysis Tool, are really general-purpose tools for comparing any given list of genes with any other list of genes. The authors of both tools suggest extending their use to comparing a user's data with 'previously published gene lists' [2], or 'comparing microarray data studying apoptosis, hypoxia, etc. with microarray data focusing on clinical backgrounds, like cancer, (viral) infections or neurological disease' [32]. L2L was conceived and developed independently of either of these tools, but fills the need that their authors, and others, have identified. Moreover, it does so in a way that is at once flexible, powerful, and extensible, yet simple enough to be accessible to every user of microarrays.

## Figures and Tables

**Table 1:** Sample data subjected to *p *value adjustment by Bonferroni correction or random-data simulation

	Actual diabetic nephropathy (downregulated) data							Random-data simulation (10,891 trials)			
	
Name of L2L database list	Total U95Av2 probes on list	Expected matches	Actual matches	Binomial *p *value	Hypergeometric *p *value	Poisson *p *value	Bonferroni-adjusted binomial *p *value	Median binomial *p *value	*p *value (list-specific) of actual binomial *p *value	*p *value (all lists) of actual binomial *p *value	FDR of actual binomial *p *value
ifn_beta_up	135	5.83	31	1.9E-14	n/a	4.3E-14	6.7E-12	0.53	<9.2E-05	<2.6E-07	<9.2E-06
ifn_alpha_up	111	4.79	28	2.7E-14	1.4E-14	6.1E-14	9.5E-12	0.52	<9.2E-05	<2.6E-07	<9.2E-06
ifn_all_up	73	3.15	19	2.0E-10	1.4E-10	1.9E-10	7.0E-08	0.61	<9.2E-05	<2.6E-07	<9.2E-06
nf90_up	59	2.55	17	3.1E-10	2.3E-10	2.9E-10	1.1E-07	0.73	<9.2E-05	<2.6E-07	<9.2E-06
ifnalpha_both_up	36	1.55	13	1.6E-09	1.3E-09	1.3E-09	5.9E-07	0.80	<9.2E-05	<2.6E-07	<9.2E-06
adip_diff_cluster2	43	1.86	12	1.8E-07	1.5E-07	9.0E-08	6.6E-05	0.56	<9.2E-05	<2.6E-07	<9.2E-06
emt_up	104	4.49	18	5.1E-07	3.4E-07	2.9E-07	1.8E-04	0.66	<9.2E-05	<2.6E-07	<9.2E-06
hpv31_dn	69	2.98	14	1.3E-06	9.6E-07	6.2E-07	4.6E-04	0.58	<9.2E-05	2.6E-07	9.2E-06
ifnalpha_either_up	83	3.58	15	2.5E-06	1.8E-06	1.2E-06	9.0E-04	0.70	<9.2E-05	1.3E-06	4.2E-05
adip_vs_fibro_up	55	2.38	12	3.2E-06	2.5E-06	1.4E-06	1.2E-03	0.69	<9.2E-05	1.5E-06	4.6E-05
hypoxia_normal_up	243	10.49	27	8.3E-06	n/a	5.5E-06	3.0E-03	0.60	9.2E-05	3.9E-06	9.8E-05
hypoxia_reg	60	2.59	12	8.5E-06	6.5E-06	3.5E-06	3.0E-03	0.74	<9.2E-05	4.4E-06	9.8E-05
tnfalpha_adip_up	17	0.73	6	5.3E-05	4.8E-05	1.2E-05	0.019	0.53	<9.2E-05	2.2E-05	3.4E-04
cmv_up	88	3.80	13	1.0E-04	7.4E-05	4.5E-05	0.037	0.53	<9.2E-05	3.8E-05	5.1E-04
vhl_normal_up	230	9.93	23	1.8E-04	n/a	1.1E-04	0.066	0.53	1.8E-04	6.9E-05	7.9E-04
dsrna_up	48	2.07	9	1.9E-04	1.5E-04	6.2E-05	0.068	0.62	2.8E-04	7.4E-05	8.2E-04
tnfalpha_tgz_adip_up	23	0.99	6	3.5E-04	3.0E-04	8.0E-05	0.12	0.64	5.5E-04	1.5E-04	1.4E-03
tgz_adip_up	26	1.12	6	7.1E-04	6.1E-04	1.7E-04	0.25	0.68	5.5E-04	2.9E-04	2.4E-03
hif1_targets	60	2.59	9	1.0E-03	7.9E-04	3.7E-04	0.37	0.74	6.4E-04	4.5E-04	3.6E-03
adip_vs_preadip_up	53	2.29	8	1.9E-03	1.4E-03	6.2E-04	0.67	0.67	1.7E-03	9.3E-04	6.2E-03
serum_fibroblast_core_dn	148	6.39	14	5.1E-03	n/a	2.5E-03	1	0.62	4.2E-03	2.4E-03	0.013
hypoxia_fibro_up	29	1.25	5	7.5E-03	6.1E-03	1.9E-03	1	0.72	7.3E-03	3.5E-03	0.018

n/a, calculation too complex to perform precisely.

**Table 2:** Sample data subjected to permutation analysis or comparison by gene symbol instead of probe ID

	10% Data permutation (10,891 trials)					Comparison by gene symbol			
	
Name of L2L database list	Binomial *p *value (actual)	Median permutation binomial *p *value	*p *value (list-specific) of actual binomial *p *value	*p *value (all lists) of actual binomial *p *value	FDR of actual binomial *p *value	Total gene symbols on list	Expected matches	Actual matches	Binomial *p *value
ifn_beta_up	1.9E-14	4.7E-12	0.10	3.2E-03	0.58	93	4.46	25	1.3E-12
ifn_alpha_up	2.7E-14	1.4E-12	0.11	3.5E-03	0.31	71	3.41	22	1.2E-12
ifn_all_up	2.0E-10	1.1E-08	0.20	0.011	0.80	48	2.30	14	3.5E-08
nf90_up	3.1E-10	2.7E-09	0.21	0.012	0.70	37	1.78	11	8.3E-07
ifnalpha_both_up	1.6E-09	2.0E-08	0.28	0.014	0.70	21	1.01	8	3.3E-06
adip_diff_cluster2	1.8E-07	1.5E-06	0.32	0.019	0.86	30	1.44	9	7.7E-06
emt_up	5.1E-07	2.4E-06	0.25	0.021	0.84	68	3.26	11	3.8E-04
hpv31_dn	1.3E-06	7.2E-06	0.30	0.022	0.79	49	2.35	9	4.8E-04
ifnalpha_either_up	2.5E-06	1.2E-05	0.30	0.025	0.83	50	2.40	9	5.6E-04
adip_vs_fibro_up	3.2E-06	2.0E-05	0.34	0.027	0.79	35	1.68	4	0.085
hypoxia_normal_up	8.3E-06	6.3E-05	0.24	0.030	0.77	168	8.06	23	6.5E-06
hypoxia_reg	8.5E-06	4.7E-05	0.35	0.031	0.74	39	1.87	9	7.7E-05
tnfalpha_adip_up	5.3E-05	5.3E-05	0.54	0.048	0.75	8	0.38	1	0.33
cmv_up	1.0E-04	4.1E-04	0.37	0.056	0.74	59	2.83	11	1.0E-04
vhl_normal_up	1.8E-04	4.6E-04	0.31	0.065	0.78	155	7.44	19	1.8E-04
dsrna_up	1.9E-04	9.7E-04	0.45	0.068	0.75	33	1.58	8	1.3E-04
tnfalpha_tgz_adip_up	3.5E-04	3.5E-04	0.54	0.079	0.78	11	0.53	1	0.42
tgz_adip_up	7.1E-04	7.1E-04	0.55	0.093	0.77	14	0.67	1	0.50
hif1_targets	1.0E-03	4.2E-03	0.46	0.10	0.80	34	1.63	6	5.2E-03
adip_vs_preadip_up	1.9E-03	7.6E-03	0.49	0.12	0.80	35	1.68	3	0.24
serum_fibroblast_core_dn	5.1E-03	0.012	0.42	0.16	0.87	114	5.47	9	0.098
hypoxia_fibro_up	7.5E-03	7.5E-03	0.62	0.17	0.86	22	1.06	3	0.086
